# National and subnational all-cause and cause-specific child mortality in China, 1996–2015: a systematic analysis with implications for the Sustainable Development Goals

**DOI:** 10.1016/S2214-109X(16)30334-5

**Published:** 2016-12-20

**Authors:** Chunhua He, Li Liu, Yue Chu, Jamie Perin, Li Dai, Xiaohong Li, Lei Miao, Leni Kang, Qi Li, Robert Scherpbier, Sufang Guo, Igor Rudan, Peige Song, Kit Yee Chan, Yan Guo, Robert E Black, Yanping Wang, Jun Zhu

**Affiliations:** a National Office of Maternal and Child Health Surveillance of China, Department of Pediatrics, West China Second University Hospital, Sichuan University, Chengdu, China; b Department of Population Family and Reproductive Health, Johns Hopkins Bloomberg School of Public Health, Baltimore, MD, USA; c The Institute for International Programs, Department of International Health, Johns Hopkins Bloomberg School of Public Health, Baltimore, MD, USA; d UNICEF China, Beijing, China; e UNICEF Regional Office for South Asia, Kathmandu, Nepal; f Centre for Global Health Research, Usher Institute of Population Health Sciences and Informatics, University of Edinburgh, Edinburgh, UK; g Nossal Institute for Global Health, University of Melbourne, Melbourne, VIC, Australia; h Peking University Health Science Center, Beijing, China; i Key Laboratory of Birth Defects and Related Diseases of Women and Children, Sichuan University, Ministry of Education, Chengdu, China

## Abstract

**Background:**

China has achieved Millennium Development Goal 4 to reduce under-5 mortality rate by two-thirds between 1990 and 2015. In this study, we estimated the national and subnational levels and causes of child mortality in China annually from 1996 to 2015 to draw implications for achievement of the SDGs for China and other low-income and middle-income countries.

**Methods:**

In this systematic analysis, we adjusted empirical data on levels and causes of child mortality collected in the China Maternal and Child Health Surveillance System to generate representative estimates at the national and subnational levels. In adjusting the data, we considered the sampling design and probability, applied smoothing techniques to produce stable trends, fitted livebirth and age-specific death estimates to natvional estimates produced by the UN for international comparison, and partitioned national estimates of infrequent causes produced by independent sources to the subnational level.

**Findings:**

Between 1996 and 2015, the under-5 mortality rate in China declined from 50·8 per 1000 livebirths to 10·7 per 1000 livebirths, at an average annual rate of reduction of 8·2%. However, 181 600 children still died before their fifth birthday, with 93 400 (51·5%) deaths occurring in neonates. Great inequity exists in child mortality across regions and in urban versus rural areas. The leading causes of under-5 mortality in 2015 were congenital abnormalities (35 700 deaths, 95% uncertainty range [UR] 28 400–45 200), preterm birth complications (30 900 deaths, 24 200–40 800), and injuries (26 600 deaths, 21 000–33 400). Pneumonia contributed to a higher proportion of deaths in the western region of China than in the eastern and central regions, and injury was a main cause of death in rural areas. Variations in cause-of-death composition by age were also examined. The contribution of preterm birth complications to mortality decreased after the neonatal period; congenital abnormalities remained an important cause of mortality throughout infancy, whereas the contribution of injuries to mortality increased after the first year of life.

**Interpretation:**

China has achieved a rapid reduction in child mortality in 1996–2015. The decline has been widespread across regions, urban and rural areas, age groups, and cause-of-death categories, but great disparities remain. The western region and rural areas and especially western rural areas should receive most attention in improving child survival through enhanced policy and programmes in the Sustainable Development Goals era. Continued investment is crucial in primary and secondary prevention of deaths due to congenital abnormalities, preterm birth complications, and injuries nationally, and of deaths due to pneumonia in western rural areas. The study also has implications for improving child survival and civil registration and vital statistics in other low-income and middle-income countries.

**Funding:**

Bill & Melinda Gates Foundation.

## Introduction

2015 marked the end of the Millennium Development Goal (MDG) era and the beginning of the implementation of the Sustainable Development Goals (SDGs). China represents a unique example of success in achieving the MDG 4 target of reducing under-5 mortality rate by two-thirds between 1990 and 2015. Based on estimates published by the UN Interagency Group on Child Mortality Estimation (UN IGME), China's under-5 mortality rate declined from 53·8 to 10·7 per 1000 livebirths at an average annual rate of reduction (ARR) of 6·5% in 1990–2015, faster than the ARR needed to achieve the MDG 4 target of 4·4%.^1^ In fact, China achieved the MDG 4 target in 2009, 6 years ahead of time. China is also one of about only a dozen countries that achieved a faster decline of mortality in neonates than in children aged 1–59 months since 1990.^1^, ^2^

In this Article, we estimated the national and subnational levels and causes of child mortality in China annually in 1996–2015, and attempted to draw implications for achievement of the SDGs for China and other low-income and middle-income countries (LMICs). We updated our approach to estimating causes of child deaths in China from the previous single-cause models based on published data^3^ to use of adjusted empirical data from the Maternal and Child Health Surveillance System (MCHSS).

**Research in context**

**Evidence before this study**

We searched multiple databases such as PubMed, Embase, ISIS Web of Knowledge, Popline, and LILACS for studies that reported causes of deaths in children younger than 5 years in China published up to Feb 12, 2015. There were no language restrictions. Twelve eligible studies that reported causes of death in China for children younger than 5 years were identified. Two studies were nationally representative, reporting causes of deaths in children younger than 5 years for some years in 1996–2010. Ten studies reported data only for selected years and subnational geographic areas. In 2015, we published the county level under-5 mortality estimates for China for the years 1996–2012. We used data from the Maternal and Child Health Surveillance System (MCHSS), censuses, surveys, surveillance sites, and disease surveillance points. We also published in 2010 causes of child mortality for China in 2008, in which input data for the estimation were based on systematic review of peer-reviewed publications.

**Added value of this study**

In this study, we updated and improved our estimates from the previous two papers on levels and causes of child mortality, specifically to 1996–2015, to present China's progress in improving child survival in the era of the Millennium Development Goals. We introduce in detail the MCHSS, which is regarded as the highest quality data source among all sources used previously. We adjusted the empirical estimates from MCHSS, with the adjustment and estimation methods being largely independent of the previous estimation approaches. We found that estimates presented in this Article agree in general with our previous estimates for the same years. Additionally, we highlighted areas where more research is needed to understand the key factors that contributed to China's progress in most of the MDG period. Our research has implications for China and other low-income and middle-income countries (LMICs) to achieve the Sustainable Development Goals.

**Implications of all the available evidence**

China has made impressive and widespread progress in improving child survival in 1996–2015, and the establishment and evolution of the MCHSS have been important in documenting this progress. Establishment of the sample registration system and its improvement over time could be useful for other LMICs that do not have quality vital and sample registration systems. However, more rigorous research is needed to fully understand the effects of China's child-survival policy and programmes implemented during this period, which could be adopted by other LMICs.

## Methods

### Study design and participants

MCHSS is a sample registration system collecting vital statistics on levels and causes of maternal and child mortality and data on congenital abnormalities. The system is designed to be nationally and regionally representative. A child mortality surveillance network was first established in 1991 by the Ministry of Health in China. In 1996, child mortality surveillance was combined with the maternal mortality and congenital abnormality surveillance networks to form MCHSS. Since then, MCHSS has been generating estimates on levels and causes of child mortality at the national and regional level.^4^, ^5^

**Figure 1:**
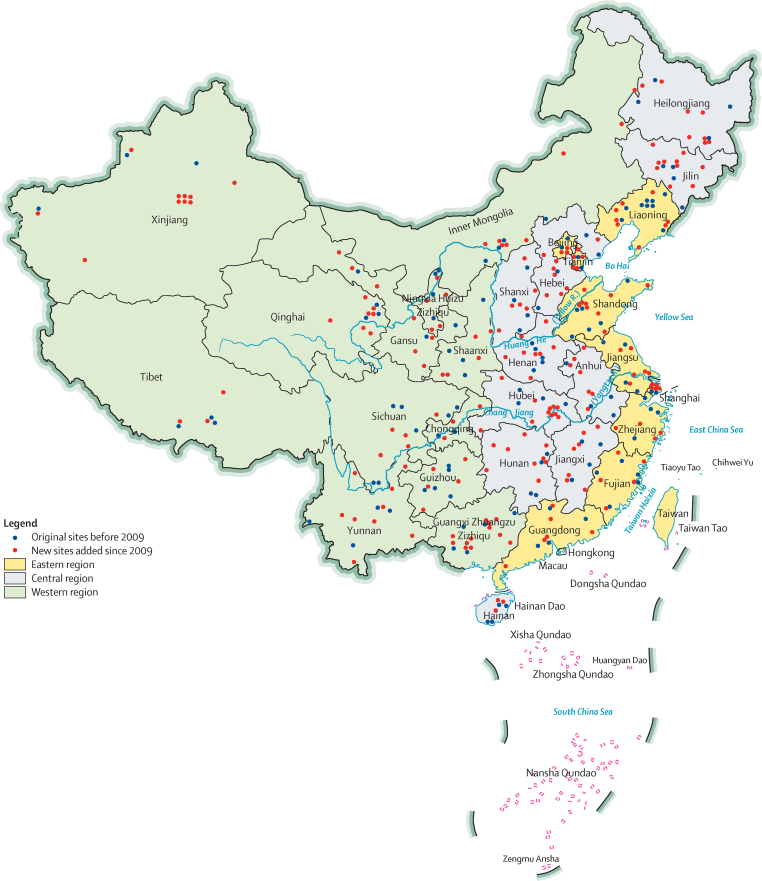
China Maternal and Child Health Surveillance System site map

In 1991, the child mortality surveillance network covered a population of 8·5 million in 81 cities or counties. When MCHSS was established in 1996, the system was expanded to 116 surveillance sites covering 37 urban districts and 79 rural counties in 31 provinces, autonomous regions, and municipalities, monitoring a total population of 12·7 million in mainland China.^6^ A major expansion of MCHSS was initiated in 2006 because of rapidly declining rates of maternal and child mortality. Training and quality assessments were done in new sites in 2006–08. Data from new sites have been incorporated since 2009. After the expansion, MCHSS came to its current structure in 2013, covering 334 sites representing 124 urban districts and 210 rural counties with a surveillance population of 47·1 million (figure 1). Additional details of the MCHSS evolution are in the appendix (p 3).

MCHSS has a multistage, stratified, clustered sampling design; stratification changed over time.^6^, ^7^ The sampling design and sample size calculation before 2006 are in the appendix (pp 4–5). When MCHSS expanded in 2006, the sample size was calculated based on infant mortality from MCHSS in 2005, crude birth rate from the National Bureau of Statistics in 2005, total population from the 2000 national census, and a design effect of 2·0 to adjust for correlation of events within the sampled areas (see appendix p 6 for more details).

The sampling was also updated in 2006. The country was divided into eastern, central, and western regions, and each region was further stratified into urban and rural areas (appendix p 7). The urban or rural classification was derived from criteria used in the 1993 National Health Services Survey and the 2006 administrative division codes published by the National Bureau of Statistics.^8^, ^9^ MCHSS is thus regarded to be representative of the six region-residency strata: eastern urban, eastern rural, central urban, central rural, western urban, and western rural areas, and of the aggregates of the six strata. The original surveillance sites remained in the sample. New sites added in 2006 were randomly sampled from urban neighbourhoods and rural townships covering 10% of the total population of the newly sampled urban districts and rural counties.

All children under 5 years of age, living in the surveillance sites and all livebirths of mothers who are either permanent residents of the sites or have lived in the sites for at least 1 year are included in MCHSS. There were no exclusion criteria. A livebirth was defined as a fetus of at least 28 weeks' gestation (or with birthweight of more than 1000 g if the gestational age was unknown), and showing any of the following signs of life after separation from his or her mother: heartbeat, breathing, umbilical cord pulsation, or voluntary muscle contraction.^6^ Livebirths were recorded by community health workers in urban areas or village doctors in rural areas and reported to neighbourhood (urban) or township (rural) Maternal and Child Health Centers (MCHCs) on a monthly basis. The information was compiled by district (urban) or county (rural) MCHCs and reported to higher (than district or county) level (city, prefecture, and province) MCHCs on a quarterly basis.

### Outcomes

The recording and reporting of deaths and causes of deaths were implemented in both communities and health facilities. Details of the reporting and cause ascertainment have been published elsewhere.^10^ Briefly, deaths occurring in communities were recorded and reported to township MCHCs within 10 days of the events by village doctors. Deaths and causes of deaths were then investigated, ascertained, and reported using the Child Death Registration Card (appendix pp 8–9) by township MCH physicians within the next 7 days. Cause ascertainment was based on death certificates of children who died in health facilities, the last clinical diagnosis if children were discharged from health facilities within 1 month of death and died on their way home or at home, or verbal autopsy if there was no contact with the health-care system (the distribution of deaths by cause ascertaining method by age and year are in the appendix [p 10]). Verbal autopsy was done using non-standardised instruments before 2012, and the 2012 WHO verbal autopsy instrument and physician review from 2012 onwards.^11^ Deaths occurring in health facilities were recorded on the Child Death Registration Card on site and reported quarterly to district or county MCHCs. Information on deaths and causes of deaths was then compiled by district or county MCHCs and crosschecked between community and facility reports for duplicates or missing records on a quarterly basis. Health-facility reports not confirmed by community reports were further investigated to ensure they were eligible MCHSS cases. Once crosschecked, deaths and causes of deaths were reported to higher level MCHCs on a quarterly basis. Since 2007, internet-based reporting has been implemented in parallel with paper-based reporting and both systems are currently in use.^6^, ^10^, ^12^

### Quality control

Coverage, completeness, and validity of reported livebirths, deaths, and causes of deaths were reviewed at the neighbourhood or township level quarterly, and at the district or county and higher levels annually. An annual quality control study is done by the MCHSS national office in two selected provinces.^10^ Provinces rotate through the quality control study over time. Three surveillance sites, including one urban and two rural sites from each province, are randomly selected. All child deaths occurring in those sites are reviewed by the MCHSS National Office. Data triangulation is done at all levels of MCHSS through cross-validation of reported births and deaths through multiple sources to identify missing events. These sources include local health facilities, family planning offices, Centers of Disease Control, and public security bureaus.^6^, ^10^, ^12^ Since 2010, the neonatal death audit has been implemented in MCHSS (see appendix p 11 for details).

Rates of under-reporting for livebirths and child deaths were calculated as the fraction of missed cases identified in the annual quality control study out of total cases captured (appendix p 11). The rate of under-reporting of livebirths was less than 1·5% in urban areas and less than 3% in rural areas in 1996–2015. Rates of under-reporting were less than 1% in the eastern region, less than 4% in the central region, and less than 3·5% in the western region. The rates of under-reporting of under-5 deaths were 10–30% at the national level, 10–25% in the eastern region, 12–35% in the central region, and 16–33% in the western region.

### Data analysis

Before 2014, information on causes of deaths was collected and grouped into 35 categories in MCHSS (appendix p 9). These categories were mapped to aggregated codes of the International Classification of Diseases 10th version (ICD-10) during the analyses (appendix pp 12–14). Since 2014, causes of deaths have been directly coded using ICD-10 in MCHSS. Causes were further mapped and aggregated to the Child Health Epidemiology Reference Group categories in this study.^13^, ^14^

In this study, MCHSS data are available annually for the period 1996–2015. We adjusted MCHSS empirical data to generate nationally and subnationally representative estimates of the numbers of deaths and mortality rates of deaths by cause. Specifically, we first adjusted the annual number of deaths by cause using a 3 year moving average of under-reporting rates by age group (0–6 days, 7–27 days, 28 days–5 months, 6–11 months, 12–23 months, and 24–59 months), type of residency (urban or rural), and region (eastern, central, or western). We calculated sampling probabilities based on best available estimate of the total population under surveillance and the region-residency census population (appendix p 15). For 1996, we based the total population on the 1990 national census, whereas the population under surveillance was based on the 1996 MCHSS. For 2000 and 2010, we based the total population on national censuses and the population under surveillance on MCHSS data in the corresponding years. The sampling probability ranged from 0·4% in rural central regions in 1996 to 6·6% in urban western regions in 2010. The sampling probabilities increased consistently in 2010, and more so in rural strata. We applied the sampling probabilities to the number of livebirths in MCHSS to derive livebirth estimates for each region-residency stratum such that the 1996 probabilities were applied to livebirths estimates for 1996–99, the 2000 probabilities were applied to 2000–09, and the 2010 probabilities were applied to 2010 onwards. We then normalised stratum-specific livebirths to the UN's estimates of livebirths for China^15^ at the national level proportionally to ensure global and regional comparability and smoothed stratum-specific proportions of livebirths using a 3 year moving average to obtain stable trends.

We calculated the total numbers of all-cause deaths by age-region-residency strata using region-residency-specific livebirths defined above, and a 3 year moving average of age-region-residency-specific all-cause mortality rates. We normalised the total numbers of stratified all-cause deaths to fit the total number of deaths of neonates and 1–59-month-old children generated by UN IGME for 1996–2015.^1^ Within each age-region-residency stratum, we smoothed the cause-specific mortality fractions using a 7 year moving average, with the highest weight given to the fourth year and the lowest and equal weights given to the first and seventh year. We then calculated the numbers of deaths by cause based on the smoothed cause-specific mortality fractions and age-region-residency-specific all-cause deaths.

Deaths due to infrequent causes, including HIV or AIDS, pertussis, malaria, and measles, were challenging to capture in MCHSS. We used WHO programme estimates for these causes instead.^2^ We derived subnational estimates for these causes assuming the number of cause-specific deaths were distributed similarly to all-cause deaths across strata.^2^ Although the assumption could be problematic for localised infectious epidemic such as malaria, the total numbers of deaths due to these conditions were rather small (eg, 3–8 malaria deaths were estimated in 1996–2015). Therefore, biases associated with this assumption are likely to be small. The numbers and fractions of all the causes were normalised to all-cause deaths within each age-region-residency stratum. Subnational estimates were aggregated to derive regional and national estimates.

### Comparison with other estimates, uncertainty estimation, and transparency

We compared our estimates with those produced by systematic analysis^16^ for the period 2009–15. The analysis^16^ was based on a systematic review of Chinese scientific literature that included all published studies meeting prespecified inclusion criteria on causes of child mortality,^16^ and was an update of our previous estimates applying single-cause models to derive cause-specific mortality fractions for the period 2000–08.^3^, ^14^ These estimates are based on nearly 300 studies published from a subset of MCHSS sites.^1^ Because input data are not exactly the same and the estimation strategies of the two approaches have limited overlap, the two sets of estimates are partially independent. We used accuracy of cause-specific mortality fractions^17^ to measure the population-level agreement between the two sets of estimates on cause distribution. Cause-specific mortality fraction accuracy is scaled between 0 and 1 (1 indicating perfect agreement).

The uncertainty of estimated deaths by cause for each year, and the uncertainty in the time trend was estimated by incorporating resampling methods of the bootstrap.^18^ The stratified total number of surveillance sites was selected at random with replacement in each region over 1000 independent instances, building a distribution for possible observed causes of deaths in each stratum. The 2·5th and 97·5th percentiles of this bootstrapped distribution were used as the uncertainty bounds for the numbers of deaths by cause.

This study conforms to the Guidelines for Accurate and Transparent Health Estimates Reporting (GATHER) statement,^19^ which promotes transparency and replicability of global health estimates (see appendix p 16 for checklist). Additional details of the input data, estimation methodology including statistical codes, and estimates are online and publicly available through the Maternal and Child Epidemiology Estimations website.

### Role of the funding source

The funder of the study had no role in study design, data collection, data analysis, data interpretation, or writing of the report. All coauthors involved in the analyses had access to the data in the study and the corresponding authors had final responsibility for the decision to submit for publication.

## Results

**Figure 2:**
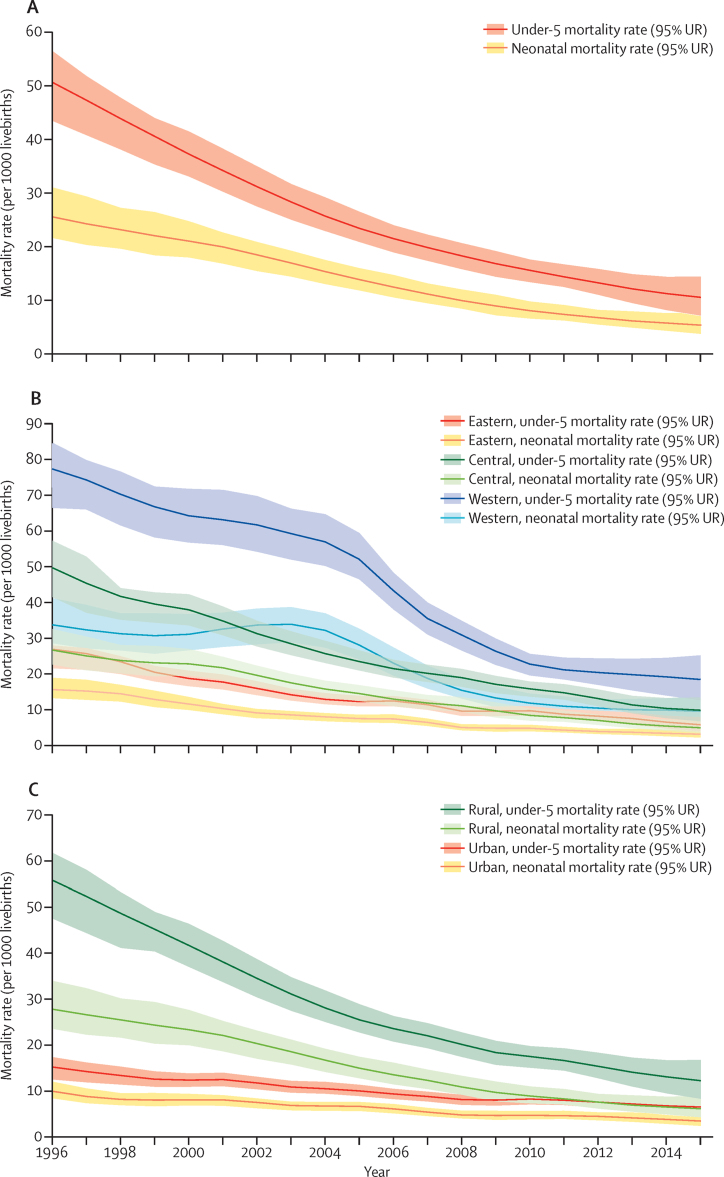
Age-specific mortality rates in children younger than 5 years in China, 1996–2015 (A) National. (B) By region. (C) By residency. UR=uncertainty range.

In 2015, 181 600 children died before the age of 5 in China, with 93 400 (51·5%, 95% uncertainty range [UR] 44·0–60·0) occurring in the neonatal period. At the national level, under-5 mortality rate fell by nearly 80% in the past two decades, from 50·8 deaths per 1000 livebirths in 1996 to 10·7 per 1000 livebirths in 2015 (figure 2A), resulting in an ARR of 8·2% per year (table 1). Neonatal mortality rate also declined substantially, from 25·7 deaths per 1000 livebirths in 1996 to 5·5 per 1000 livebirths in 2015. The rate of decline was faster in the first decade than in the second decade for under-5 mortality rate, but steadier for neonatal mortality rate (figure 2A).

Child mortality rates showed large variation by region in 1996–2015 (figure 2B). The western region had the highest under-5 mortality rate and neonatal mortality rate in 2015, with an estimated under-5 mortality rate of 18·5 (95% UR 12·6–25·2) deaths per 1000 livebirths and neonatal mortality rate of 9·5 (6·8–13·7) per 1000 livebirths. The under-5 mortality rate in the eastern region was 5·8 deaths per 1000 livebirths and neonatal mortality rate in the eastern region was 3·1 per 1000 livebirths, similar to the rates in the USA and Canada. The geographical inequalities, represented by the ratio of under-5 mortality rate and neonatal mortality rate between western and eastern regions, fluctuated but generally increased during this period. For example, the ratio of under-5 mortality rate increased from 2·9:1 in 1996 to 4·4:1 in 2004, dropped to 2·3:1 in 2005–2010, then increased again to 3·2:1 in 2015. Since 1996, under-5 mortality rate in all three regions have been decreasing with an ARR of 7·5% or greater; the central region had the fastest ARR of 8·5% (95% UR 6·8–10·8; table 1).

Inequity in child mortality between rural and urban areas was also apparent in China in 1996–2015 (figure 2C). In 2015, 81·1% (95% UR 80·9–81·4) of under-5 deaths occurred in rural China. This proportion reduced from 96·2% (93·5–96·5) in 1996, which is probably reflective of rapid urbanisation. As a result, despite a continued reduction in under-5 mortality rate in urban areas (by 56·5% [39·5–68·2] between 1996 and 2015), the actual number of under-5 deaths occurring in urban areas increased by 27·2% (9·7–40·6). In rural areas, mortality rates fell by more than 75% for neonates (77·5% [68·1–82·4]) and children aged under-5 years (77·8% [67·9–80·2]).

**Figure 3:**
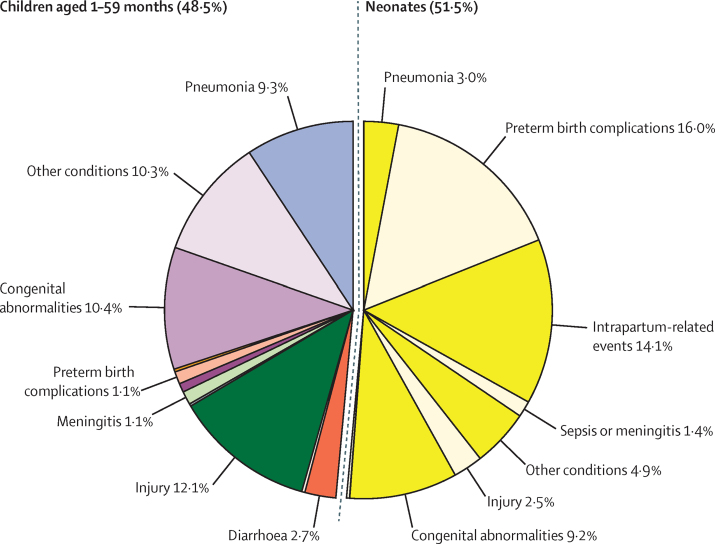
Causes of deaths in children aged 1–59 months and neonates in China, 2015 Causes of death that caused less than 1% of all deaths are not labelled.

The leading causes of under-5 mortality in 2015 in China were congenital abnormalities (19·7% [95% UR 18·1–21·5]), preterm birth complications (17·0% [14·9–19·7]), and injuries (14·6% [13·4–15·8]) (figure 3; table 2). The leading causes in neonates were preterm birth complications (16·0% [13·7–18·9] of under-5 deaths), intrapartum-related events (14·1% [12·1–16·6]), and congenital abnormalities (9·2% [8·0–11·1]). Among children aged 1–59 months, the leading causes were injuries (12·1% [10·6–13·5]), congenital abnormalities (10·4% [8·0–12·6]), and pneumonia (9·3% [6·9–11·0]).

**Figure 4:**
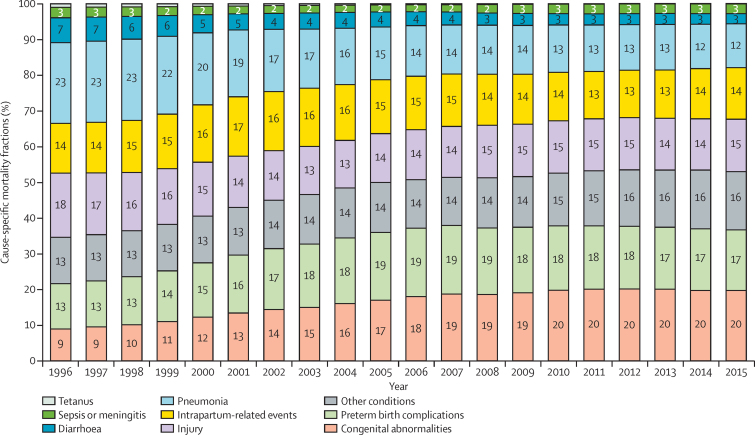
Cause of mortality in children younger than 5 years in China, 1996–2015

The contribution of major infectious causes, such as pneumonia and diarrhoea, to all-cause under-5 deaths decreased from 1996 to 2015 (figure 4). Pneumonia was the leading cause of death in 1996, accounting for 22·6% (95% UR 20·8–25·3) of all-cause under-5 deaths (table 2). The contribution of pneumonia dropped to 12·2% (10·1–13·8), making it the 6th leading cause in 2015. The proportion of deaths due to diarrhoea decreased from 6·9% (5·2–7·8) in 1996 to 2·9% (2·1–3·5) in 2015. Conversely, the proportions of under-5 deaths due to congenital abnormalities increased from 9% (8·1–11·0) in 1996 to 20% (18·1–21·5) in 2015. Congenital abnormalities have become the leading cause of under-5 deaths since 2009. The contribution of preterm birth complications and intrapartum-related events were more stable in this period, ranging between 12·7% (12·4–15·7) and 19·2% (17·4–21·0).

In 1996–2015, cause-specific mortality rates decreased with striking, albeit varying ARRs (table 1). The causes of death with the most rapid decline in children younger than 5 years were infectious diseases, including neonatal tetanus, diarrhoea, and pneumonia, with ARRs of greater than 10% at the national level and across regions. All other causes decreased at ARRs greater than that needed to achieve MDG 4 (4·4%), with the exception of congenital abnormalities. The reduction of mortality due to congenital abnormalities was particularly slow in the central region (ARR 3·5% [95% UR 1·4–6·2]) and the western region (2·6% [0·0–6·2]).

The cause-of-death composition also varied across age groups. Distributions of age-specific causes-of-death are shown in the appendix (p 18). Intrapartum-related events, preterm birth complications, and congenital abnormalities were the leading causes in the early neonatal period (0–6 days). Collectively, they accounted for over three-quarters of all early neonatal deaths. For deaths occurring in the late neonatal period (7–27 days), pneumonia replaced intrapartum-related events and ranked among the top three leading causes. Congenital abnormalities, pneumonia, and injuries were the leading causes of death in children who died at the ages of 28 days–5 months and 6–11 months. After the first year of life, injuries became the leading cause, contributing 41·1% (95% UR 38·8–42·9) of deaths in children aged 12–23 months and 53·8% (51·4–57·5) of deaths in children aged 24–59 months. The relative importance of preterm birth complications dropped sharply across the six age groups once children had survived the neonatal period. The contribution of congenital abnormalities to mortality remained high through infancy, after which it declined. Injuries started to gain prominence after the neonatal period and became dominant after the first year of life (appendix p 18).

The cause distribution was also somewhat different between rural and urban areas (appendix p 19). In rural areas, congenital abnormalities and preterm births were followed by injuries as the leading causes, whereas in urban areas intrapartum-related events were ranked third. The distribution of causes of under-5 deaths also differed by region (appendix p 19). Whereas congenital abnormalities, preterm birth complications, and injuries were the leading causes in the eastern and central regions, pneumonia caused more deaths than did injuries in the western region.

**Figure 5:**
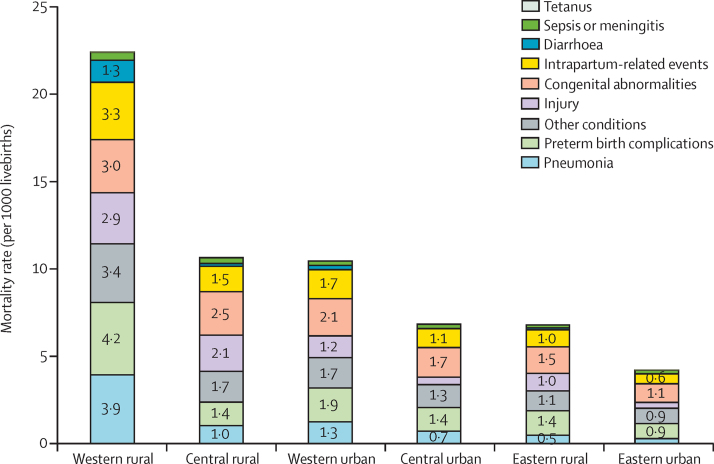
Cause-specific mortality rate in children younger than 5 years by region-residency strata in China, 2015 Causes with mortality rate of less than 0·5 per 1000 livebirths are not labelled.

The western rural area is the least developed area of the six region-residency strata. Under-5 mortality rate in the western rural area was estimated to be 22·4 (95% UR 18·1–28·1) per 1000 livebirths in 2015, at least twice that of under-5 mortality rate in other region-residency strata (figure 5). The western rural area was also the only stratum with an infectious disease (pneumonia) as the leading cause, whereas congenital abnormalities were the leading cause in all other strata. Injuries contributed to higher mortality rates in rural areas than urban areas across all regions. The gap in mortality rates between western rural and eastern urban areas widened for major non-infectious causes in 1996–2015. For example, the ratio of mortality rates due to congenital abnormalities between the two areas increased from 1·2 in 1996 to 2·8 in 2015, and the ratio of intrapartum-related events increased from 4·1 in 1996 to 5·9 in 2015.

Our MCHSS based cause-specific mortality fractions estimates for 2015 in children younger than 5 years were similar to those derived by Song and colleagues^16^ (appendix p 20). The accuracy of the cause-specific mortality fractions was estimated to be 0·92. One exception was the fraction of congenital abnormalities, which was estimated to be 20% in this study compared with 14% by Song and colleagues.^16^ More discrepancies are noted in the neonatal period; despite overall good agreement between the two sets of estimates (cause-specific mortality fractions accuracy of 0·96), the fraction of congenital abnormalities was greater in our study than that in Song and colleagues' study (18% *vs* 11%). In children aged 1–59 months, the agreement was good (cause-specific mortality fractions accuracy is 0·94), with similar fractions of major causes, including injuries, congenital abnormalities, pneumonia, and diarrhoea.

## Discussion

China has made remarkable strides in improving child survival in 1996–2015. It has achieved an impressive average annual rate of decline of under-5 mortality rate of 8·2% compared with the global figure of 3·7% in this period. Based on our estimates, sufficient progress (ie, at an ARR of at least 4·4% per year to achieve the MDG 4 target) has been achieved across regions, residency, age groups, and cause categories.

The progress coincided with China's widespread socioeconomic development in the past two decades.^20^, ^21^ This development includes, for example, the improvement in girls' education through achievement of 9 year compulsory education for all and narrowing the gender gap in years of education from 1·3 years in 2000 to 0·8 years in 2014.^20^, ^21^ National commitment to the improvement of maternal and child survival and health could be another important contributor, manifested by strengthened strategic legislation (eg, the Law on Maternal and Infant Health Care) and institutionalised policy frameworks (eg, the National Action Plan for the Development of Children).^20^, ^21^ Rigorous quantitative and qualitative research is still needed to understand the mechanisms through which these national development and action plans have been successful.

China officially eliminated neonatal tetanus in 2012.^22^ Despite not having a national programme on maternal tetanus toxoid immunisation, this success was credited to increased institutional delivery through the Safe Motherhood Initiative and the national Program to Reduce Maternal Mortality and Eliminate Neonatal Tetanus to improve local obstetric infrastructure, establish a fast-channel referral mechanism for pregnant women at labour, subsidise institutional delivery, and offer community-based health education.^20^, ^23^ More research is needed to establish the contribution of economic development in addition to these national programmes.^24^ China outperformed most of the 74 Countdown to 2015 countries (countries with high under-5 mortality rate, large numbers of under-5 deaths, or both) in its progress on major infectious causes, such as pneumonia and diarrhoea.^25^ Improved socioeconomic status, water, sanitation, nutrition, and access to integrated management of childhood illness could be contributors to success.^10^, ^26^, ^27^ Major causes of neonatal mortality, such as preterm birth complications and intrapartum-related events, have also been steadily declining, which might be associated with increased quality of institutional delivery and neonatal resuscitation.^28^, ^29^ Although existing research has implied causal relationships between some of these interventions and cause-specific mortality rates, more rigorous evaluation studies that can adequately establish causal relationships are needed.

Despite the progress, 181 600 children died before their fifth birthday in China in 2015. Major causes of child deaths included congenital abnormalities, preterm birth complications, and injuries. The list of leading causes is similar to that in high-income countries, such as the USA.^30^ Congenital abnormalities are not only the leading cause, but also one that becomes increasingly important. Previous studies suggest that integrated preventive and treatment strategies combining political commitment, folic acid supplementation, and improved insurance coverage of surgeries addressing congenital heart diseases could be effective.^31^, ^32^, ^33^ Staple food fortification with folic acid could also be considered.^20^, ^32^ Primary and secondary prevention of preterm birth complications and injuries should receive more attention from the medical community, the government, and the general public. Effective interventions could include antenatal corticosteroids and kangaroo mother care for preterm births, and seat-belts and helmets to prevent road traffic injuries.^34^, ^35^ More research is needed to further understand the effectiveness of these interventions in the Chinese context and, if effective, how to implement these interventions at scale.^2^

Despite a similar pace of decline across regions, almost half of all deaths in children younger than 5 years occurred in the least developed western region in 2015. The mortality rate in the western region was more than three times that of the most developed eastern region, and this gap increased by 10% since 1996. Overall, inadequate health service infrastructures and low health expenditure due to economic underdevelopment are thought to have resulted in limited access and use of health services in mothers and children, which led to inequity in child survival status in the western region.^36^, ^37^ Although the quantity of MCH workers did not seem to be an issue in this region, their education level was worse than that in the other two regions.^38^ This might have affected the quality of child health services provided. Investment in strategies to encourage the flow of better-educated and well trained MCH staff to the western region could be a policy option. MCH leadership and champions are also needed to improve child survival in the less developed areas. Clearly, the regional disparities in child mortality need to be systematically addressed in the SDG era. Child survival policy, programmes, and resources targeting the western region should receive more attention.

With 80% of under-5 deaths in 2015, rural areas should be another focus for child survival in the SDG era. Several rural-focused national policies and programmes, such as the Program to Reduce Maternal Mortality and Eliminate Neonatal Tetanus and the Safe Motherhood Initiative, have been in place since the early 2000s through enhanced subsidies of institutional delivery in rural women. These programmes might have contributed to the steady decline of child mortality in rural China and more evidence is needed to support this claim.^20^ Ensuring continued success of these programmes is crucial, especially in hard-to-reach areas where child mortality is probably the highest. Rapid urbanisation has been shifting the burden of deaths in children younger than 5 years from rural to urban areas, yet the survival, health, and wellbeing of migrant children or children of migrant parents who are left behind are poorly understood.^39^ Injuries and pneumonia should receive more attention from the central and local government, the medical and public health community, and the general public in rural areas whereas congenital abnormalities and preterm birth complications are the priorities in urban areas.

More than half of under-5 deaths in China in 2015 were of neonates. Based on the estimated age-specific cause-of-death distributions, interventions targeting intrapartum-related events should focus on the early neonatal period. Programmes for preterm birth complications need to target the entire first month of life. Interventions addressing congenital abnormalities are important throughout the first year of life. After the age of 1 year, child survival policy and programmes should clearly concentrate on preventing and treating injuries. Research to obtain similar understanding of the age-specific cause composition is needed in other LMICs to help policy makers and programme managers to better prioritise and allocate scarce resources by age.

Our data source, MCHSS, has been growing and evolving since its establishment. Reporting completeness has improved over time and the proportion of causes ascertained by medical certification has increased, aided in part by increased use of hospitals.^40^, ^41^ Most deaths ascertained by verbal autopsy were due to injuries, which are relatively accurately identified by this method.^42^ Increasing completeness suggests that surveillance capability and data quality might have improved. However, the pace of improvement might not have been even. For example, neonatal mortality rate in the western region increased in 2000–03 before resuming the downward trend in 2004 (figure 2B). This small peak in the trend line might not be real, given the rapid decline in neonatal mortality rate in other regions and in the national under-5 mortality rate. Instead, the increase in mortality rate observed during that period could reflect improved surveillance capacity in detecting neonatal deaths after 2000 in the western region.

Our study has some limitations. First, MCHSS considered local capacity when sampling prefectural-level urban cities and rural counties, which could have introduced biases. Second, the sampling probabilities were calculated using total population under surveillance and region-residence-stratum-specific census population estimates that are the best available. Because a census is only done every 10 years and China has been experiencing rapid urbanisation, the sampling probabilities are approximations. Third, the definition of livebirths was restricted to those born after 28 weeks of gestation, which could underestimate neonatal and under-5 mortality rates and the contribution of major neonatal causes, especially preterm birth complications. Given the relatively small burden of those born alive before 28 weeks, this bias is likely to be small. We did a sensitivity analysis comparing estimates based on the narrow definition of livebirth and that of all livebirths in selected sites where information on both was collected and found only small differences (data not shown). A plan is being developed to update the livebirth definition in MCHSS. This update should be easier to implement now because China has officially adopted the two-child policy. Lastly, given the nature of sample surveillance systems in which coverage is overall low at the national level, trends based on raw data are not smooth (appendix p 21). We have chosen to smooth the raw data and fit to the UN IGME estimates. The process might have inadvertently masked real short-term trends.

Despite the additional adjustments performed, however, MCHSS raw estimates fall within the uncertainty of UN IGME estimates for most years (appendix p 21). We also published in 2014 the national trends of under-5 mortality rate and neonatal mortality rate for China for the period 2000–12.^43^ The under-5 mortality rate estimates agree well with those produced by UN IGME, but the neonatal mortality rate estimates are significantly lower than those of UN IGME in 1996–2005 and start to converge to the UN IGME estimates after 2006. In the 2014 paper, input data from MCHSS and other sources were used, including vital registration and maternal and child annual reporting. Estimation methods were also different from those used in the UN IGME. That the two sets of relatively independent estimates agree reasonably well in 10 years is encouraging. Similarly, our estimates of cause-of-death distributions generally agree with estimates produced by Song and colleagues.^16^

China's Western Development is arguably one of the biggest development initiatives in the world. With a combination of socioeconomic development and regional MCH programmes, such as the Program to Reduce Maternal Mortality and Eliminate Neonatal Tetanus which was first piloted regionally in 2000,^20^ the western region has had great progress in improving child survival. Lessons learnt in this process, particularly those assessed by rigorous evaluations, are probably the most relevant to other LMICs. However, not all of China's experiences are readily transferrable to other countries and context. For example, institutional delivery rate has increased by about 30% in urban areas (76·5% in 1996 to 99·7% in 2012) and 91% in rural areas (51·7% in 1996 to 98·8% in 2012).^44^ These increases might have contributed to China's advances in child, and particularly neonatal, survival. But such a rapid increase in institutional delivery rate could be challenging to replicate in other LMICs. In addition to coverage, quality of institutional delivery rate is also a key consideration. For low-income settings not ready for the scale-up of institutional delivery rate, community-based neonatal interventions, such as kangaroo mother care and chlorhexidine cord cleansing,^45^ could be suitable options.

The experiences of establishing and improving MCHSS could also prove informative to other LMICs where adequate vital or sample registration system is unavailable or of low quality. Where access to health care is high, a dual system of collecting vital events through community and health facilities can be attempted, as was done in MCHSS, so that crosschecking can be routinely implemented during quality control. Other forms of data triangulation across sectors and routine quality control mechanisms are also possible lessons from which other LMICs could learn.

We consider our transition from model-based estimates to estimates based on empirical data for China an important step forward. This approach not only helps improves the validity of our estimates, but also increases the use of sample registration system data for national policy making and international exchange. Our study shows that strengthened civil registration and vital statistics could support and inform health policy and planning and help global health debates. We hope that China's experiences of developing MCHSS and achieving rapid improvement of child survival can contribute to the discussion on strengthening civil registration and vital statistics and on continued investment in child survival in LMICs in the SDG era.

For the **Maternal and Child Epidemiology website** see http://tinyurl.com/Hopkins-MNCH-Chinacod-openacce

## Figures and Tables

**Table 1:** National and regional average annual rate of reduction and uncertainty range of all-cause mortality rates and cause-specific mortality rates in children younger than 5 years in China, 1996–2015

	**National**	**Eastern region**	**Central region**	**Western region**
**All cause**				
Under-5 mortality rate	8·2% (6·4–9·7)	8·1% (6·4–9·6)	8·5% (6·8–10·8)	7·5% (5·8–9·0)
Under-5 mortality rate in urban areas	4·4% (2·8–6·0)	6·0% (4·4–7·6)	4·1% (2·6–5·8)	4·5% (2·8–6·1)
Under-5 mortality rate in rural areas	7·9% (6·2–9·4)	7·9% (6·2–9·4)	8·6% (6·8–10·0)	6·7% (5·0–8·2)
Early neonatal mortality rate	8·4% (5·0–9·1)	8·6% (6·9–11·0)	9·4% (7·7–11·8)	6·7% (5·0–9·1)
Late neonatal mortality rate	7·1% (4·9–9·0)	7·9% (6·2–10·3)	7·0% (5·3–9·4)	6·6% (4·9–9·0)
Neonatal mortality rate	8·1% (6·4–10·5)	8·5% (5·9–10·8)	8·9% (7·2–11·3)	6·6% (5·0–9·0)
Infant mortality rate	7·2% (5·3–8·6)	7·1% (5·9–9·2)	7·7% (6·5–9·8)	6·5% (5·3–8·6)
**By cause**				
Neonatal tetanus	26·4% (22·2–39·3)	23·7% (8·6–29·8)	34·0% (9·4–42·7)	20·8% (16·4–30·5)
Diarrhoea	12·8% (10·1–15·7)	12·9% (9·3–19·1)	15·5% (11·7–19·9)	11·7% (8·8–14·9)
Pneumonia	11·4% (9·7–13·7)	12·5% (9·4–16·7)	12·4% (10·5–15·0)	10·3% (8·3–12·8)
Measles	9·4% (6·9–14·1)	8·7% (5·8–13·1)	9·2% (6·7–13·9)	9·4% (8·0–15·3)
Injury	9·3% (5·4–9·6)	9·2% (5·8–10·4)	9·3% (5·2–9·6)	8·5% (3·7–10·2)
Intrapartum-related conditions	8·6% (6·3–10·5)	8·5% (5·6–10·2)	8·6% (7·3–11·5)	8·1% (4·3–9·1)
Sepsis or meningitis	8·0% (5·9–10·6)	7·6% (5·0–11·9)	9·1% (5·5–11·0)	6·4% (4·4–11·0)
Preterm birth complications	6·6% (5·0–9·1)	6·9% (5·2–9·5)	8·3% (6·6–10·9)	4·5% (2·6–7·2)
Congenital abnormalities	4·1% (2·3–6·1)	6·5% (4·6–9·1)	3·5% (1·4–6·2)	2·6% (0·0–6·2)
Other conditions	7·0% (5·9–9·7)	6·8% (4·6–8·7)	6·7% (5·8–9·3)	7·0% (5·5–9·9)

Data are annual rate of reduction (95% uncertainty range).

**Table 2:** Estimated numbers of child deaths and mortality rates by cause in China in 1996 and 2015

	**1996**		**2015**	
	Estimated number of deaths	Cause-specific mortality rate (per 1000 livebirths)	Estimated number of deaths	Cause-specific mortality rate (per 1000 livebirths)
**Children aged 0–59 months**				
Congenital abnormalities	62 700 (50 200–69 800)	4·5 (3·6–5·1)	35 700 (28 400–45 200)	2·1 (1·7–2·7)
Preterm birth complications	88 800 (76 500–101 200)	6·4 (5·5–7·3)	30 900 (24 200–40 800)	1·8 (1·4–2·4)
Injury	126 100 (83 400–146 700)	9·1 (6·0–10·6)	26 600 (21 000–33 400)	1·6 (1·3–2·0)
Intrapartum-related events	97 600 (84 600–111 400)	7·1 (6·1–8·1)	26 100 (20 500–34 600)	1·5 (1·2–2·0)
Pneumonia	158 200 (129 800–163 000)	11·5 (9·4–11·8)	22 200 (15 900–28 000)	1·3 (0·9–1·6)
Diarrhoea	48 700 (32 300–49 400)	3·5 (2·3–3·6)	5300 (3400–6900)	0·3 (0·2–0·4)
Sepsis or meningitis	20 600 (14 200–20 800)	1·5 (1·0–1·5)	5000 (3800–6500)	0·3 (0·2–0·4)
Other conditions	97 900 (70 400–111 400)	7·1 (5·1–8·1)	29 700 (24 200–40 800)	1·7 (1·4–2·3)
**Neonates aged 0–27 days**				
Preterm birth complications	85 800 (73 700–97 900)	6·2 (5·3–7·1)	29 000 (22 600–38 800)	1·7 (1·3–2·3)
Intrapartum-related events	97 500 (84 400–111 200)	7·1 (6·1–8·1)	25 700 (20 000–34 100)	1·5 (1·2–2·0)
Congenital abnormalities	36 200 (29 900–42 800)	2·6 (2·2–3·1)	16 800 (13 100–22 700)	1 (0·8–1·4)
Pneumonia	56 600 (48 100–66 000)	4·1 (3·5–4·8)	5400 (4100–7200)	0·3 (0·2–0·4)
Injury	32 500 (26 200–40 100)	2·4 (1·9–2·9)	4600 (3600–6200)	0·3 (0·2–0·4)
Sepsis or meningitis	5600 (4000–7400)	0·4 (0·3–0·5)	2500 (1800–3500)	0·1 (0·1–0·2)
Diarrhoea	2700 (2100–3400)	0·2 (0·1–0·2)	500 (300–600)	0 (0–0)
Tetanus	6500 (4100–9100)	0·5 (0·3–0·7)	..	..
Other conditions	31 200 (25 800–36 600)	2·3 (1·9–2·7)	9000 (6900–12 200)	0·5 (0·4–0·7)
**Children aged 1–59 months**				
Injuries	93 600 (73 500–113 600)	6·8 (5·3–8·2)	22 000 (16 700–27 800)	1·3 (1·0–1·6)
Congenital abnormalities	26 600 (18 300–29 300)	1·9 (1·3–2·1)	19 000 (13 000–25 100)	1·1 (0·8–1·5)
Pneumonia	101 600 (75 100–104 000)	7·4 (5·4–7·5)	16 800 (11 100–21 700)	1·0 (0·7–1·3)
Diarrhoea	45 900 (30 000–46 600)	3·3 (2·2–3·4)	4800 (3100–6300)	0·3 (0·2–0·4)
Meningitis	14 600 (8 500–15 100)	1·1 (0·6–1·1)	2100 (1500–2800)	0·1 (0·1–0·2)
Preterm birth complications	3000 (2200–3800)	0·2 (0·2–0·3)	1900 (1300–2500)	0·1 (0·1–0·1)
Pertussis	2200 (0–10 200)	0·2 (0·0–0·7)	1400 (1300–1400)	0·1 (0·1–0·1)
Measles	2400 (1500–3100)	0·2 (0·1–0·2)	500 (300–900)	0·0 (0·0–0·1)
Intrapartum-related events	100 (100–200)	0·0 (0·0–0·0)	400 (300–600)	0·0 (0·0–0·0)
HIV/AIDS	300 (200–600)	0·0 (0·0–0·0)	400 (200–800)	0·0 (0·0–0·0)
Malaria	..	..	..	..
Other conditions	55 800 (36 300–64 700)	4·0 (2·6–4·7)	18 800 (13 700–24 400)	1·1 (0·8–1·4)

Data are n (95% UR) or rate (95% UR). Other conditions in children aged 1–59 months included causes originating during the perinatal period, cancer, severe malnutrition, and other specified causes. Intrapartum-related events were previously referred to as birth asphyxia. UR=uncertainty range. Blank cells indicate that less than 100 deaths were estimated for the condition.
