# Supplementary material for: National and subnational all-cause and cause-specific child mortality in China, 1996–2015: a systematic analysis with implications for the Sustainable Development Goals
Source: Lancet Glob Health. 2016 Dec 20;5(2):e186–97. doi: 10.1016/S2214-109X(16)30334-5 (PMC5250590; doi:10.1016/S2214-109X(16)30334-5)
Supplement: Supplementary appendix [file mmc1.pdf]

THE LANCET Global Health

Supplementary appendix

This appendix formed part of the original submission and has been peer reviewed. We post it as supplied by the authors.

Supplement to: He C, Liu L, Chu Y, et al. National and subnational all-cause and cause-specific child mortality in China, 1996–2015: a systematic analysis with implications for the Sustainable Development Goals. *Lancet Glob Health* 2016; published online Dec 19. http://dx.doi.org/10.1016/S2214-109X(16)30334-5.

National and subnational levels and causes of child mortality in 1996-2015 in China: implications for the Sustainable Development Goals

Chunhua He, MD[1*], Li Liu, PhD [2,3*‡], Yue Chu, MSPH [3], Jamie Perin, PhD [3], Li Dai, PhD [1], Xiaohong Li, MD [1], Lei Miao, BA [1], Leni Kang, PhD [1], Qi Li, MD [1], Robert Scherpbier, MPH [4], Sufang Guo, MD [5], Prof Igor Rudan, MD [6], Peige Song, MSc [6], Kit Yee Chan, PhD [6,7], Prof Yan Guo, MPH [8], Prof Robert E Black, MD [3], Prof Yanping Wang, MD [1‡], Prof Jun Zhu, MD [1,9‡]

[1]National Office of Maternal and Child Health Surveillance of China, Department of Pediatrics, West China Second University Hospital, Sichuan University, Chengdu, China
[2]Department of Population Family and Reproductive Health, Johns Hopkins Bloomberg School of Public Health, Baltimore, USA
[3]The Institute for International Programs, Department of International Health, Johns Hopkins Bloomberg School of Public Health, Baltimore, USA
[4]UNICEF China, Beijing, China
[5] UNICEF Regional Office for South Asia, Kathmandu, Nepal
[6] Centre for Global Health Research, Usher Institute of Population Health Sciences and Informatics, University of Edinburgh, Edinburgh, Scotland, United Kingdom
[7]Nossal Institute for Global Health, University of Melbourne, Melbourne, Australia
[8]Peking University Health Science Center, Beijing, China
[9]Key Laboratory of Birth Defects and Related Diseases of Women and Children (Sichuan University), Ministry of Education, Chengdu, Sichuan, China

[*]indicates joint first-authorship
[‡]indicates joint corresponding authorship

Correspondence to:
Dr. Li Liu, Department of Population, Family and Reproductive Health, and the Institute for International Programs, Department of International Health, Johns Hopkins Bloomberg School of Public Health, 615 N Wolfe Street, Baltimore, MD 21205, USA lliu26@jhu.edu

Prof Yanping Wang, National Office of Maternal and Child Health Surveillance, West China Second University Hospital, No.20, Section 3, Renmin South Road, Chengdu, 610041 China wyxyanping@163.com

Prof Jun Zhu, National Office of Maternal and Child Health Surveillance, West China Second University Hospital, No.20, Section 3, Renmin South Road, Chengdu, 610041 China, zhu_jun1@163.com

Supplementary webappendix

Webappendix 1. Evolution of the China Maternal and Child Health Surveillance System (MCHSS)

Webappendix 2. The sampling design of MCHSS before 2006

Webappendix 3. Additional details on sample size calculation of MCHSS

Webappendix 4. MCHSS sampling

Webappendix 5. Child Death Registration Card

Webappendix 6. The distribution of deaths by cause ascertaining method by age and year, MCHSS, China

Webappendix 7. Additional details on quality control of MCHSS

Webappendix 8. Causes of deaths categorization and mapping between MCHSS classification, ICD-10, and the CHERG classification

Webappendix 9. Sampling probability (%) by region-and-residency strata in MCHSS

Webappendix 10. Guidelines for Accurate and Transparent Health Estimates Reporting (GATHER) checklist

Webappendix 11. Cause specific mortality fractions by age among children under-five years of age in China in 2015

Webappendix 12. Cause specific mortality fractions by residency and region in China in 2015

Webappendix 13. Comparison of age-and-cause-specific mortality fractions in 2015 between He et al. (our estimates) and Song et al.

Webappendix 14. Comparison of trends in national U5MR and NMR by estimation sources, China, 1996-2015

Webappendix 1. Evolution of the China Maternal and Child Health Surveillance System (MCHSS)

**Year**	**1991**	**1996**	**2001**	**2006/07**	**2009**	**2013**
Event	Child mortality surveillance network established	Child mortality, maternal mortality, and congenital abnormality surveillance networks combined	Site update[1]	MCHSS expansion initiated[2]	MCHSS expansion completed[2]	Site update[3]
Number of sites under surveillance	81	116	116 (with 17 sites from 14 provinces replaced)	123 with 13 sites updated[4]	336	334
Number of urban districts under surveillance	25	37	37 (2 sites replaced)	56 (12 switched from rural sites)	126	124
Number of rural counties under surveillance	56	79	79 (15 sites replaced)	67 (1 switched from urban site)	210	210
Number of population under surveillance (millions)	8.5	12.7	14.0	16.7	44.9	47.1

[1]To account for economic development, and changes in administrative boundaries and urban-rural classification while maintaining the total number of population under surveillance, 17 and 2 surveillance sites were re-selected in 2001 and 2009, respectively.

[2]Expansion in surveillance sites was not reflected in the data until 2009.

[3]Total number of sites changed from 336 to 334 due to site combination. Population under surveillance did not change.

[4]The changes in urban/rural classification for 13 sites were due to administrative changes for these areas based on administrative area codes published by National Bureau of Statistics of China in 2007. Before 2009, the unit of surveillance was municipalities/cities/counties. After 2009, it changed to districts/counties.

Webappendix 2. The sampling design of MCHSS before 2006

In 1991, child mortality surveillance sites were selected using a multi-stage sampling.[1,2] First, all provinces in China were stratified into three regions, namely,

- Costal Region, including Beijing, Tianjin, Shanghai, Liaoning, Shandong, Jiangsu, Zhejiang, Fujian, and Guangdong;
- Inland Region, including Jilin, Hebei, Henan, Shanxi, Anhui, Hubei, Hunan, Guangxi, Shaanxi, Jiangxi, Hainan, Heilongjiang, and Eastern Sichuan; and
- Remote Region, including Neimenggu, Ningxia, Gansu, Xinjiang, Qinghai, Yunnan, Guizhou, Xizang, and Western Sichuan.

The stratification was based on geographic location, economic status and infant mortality. All urban districts and rural counties within each region were further stratified into six strata, namely 1) large city, 2) medium/small city, 3) rural county type I, 4) rural county type II, 5) rural county type III, and 6) rural county type IV, based on health and social-economic status of the 1982 national census. Seventeen strata were established as there were no type IV rural counties in the Coastal Region.

Population under surveillance within each stratum was calculated based on stratum-specific infant mortality and crude birth rates as detailed in Webappendix 3. Prefectural-level cities and rural counties were sampled according to the following criteria to obtain representative sample within each stratum: 1) the number of cities/counties sampled with probability proportional to the number of cities/counties in each stratum, and a minimum of two cities/counties; 2) sampled cities/counties were distributed evenly across all 30 provinces considering feasibility; 3) weighted mean infant mortality of study sample was close to that of study population within each stratum; and 4) selected cities/counties had adequate human and financial resources and capability to manage and maintain surveillance sites. The resulting number of surveillance cities and counties by stratum in 1991 are shown in the table below. The total population selected exceeded the minimum sample size needed, only one district within each sampled city and two to five townships within each sampled county were sampled by systematic sampling at the end.[1] Within each stratum, the weighted infant mortality was close to the empirical stratum-specific infant mortality and the sample was considered nationally representative.[1]

Table. Number of surveillance cities/counties by stratum in 1991[1]

	**Large city**	**Small city**	**County I**	**County II**	**County III**	**County IV**	**Total**
Costal Region	5	5	6	3	3		22
Inland Region	2	9	6	10	9	2	38
Remote Region	2	2	2	4	8	3	21
Total	9	16	14	17	20	5	81
Average population per city/county (thousands)	150-300	80-150	55	50	40	30	

The total surveillance sites increased from 81 cities/counties in 1991 to 116 sites representing 123 cities/counties in 1996, with one to two cities/counties added in each province/autonomous region/municipality. In 1996-2009, the unit of surveillance was municipalities/cities in urban areas and counties in rural areas. In 2001, 17 sites from 14 provinces were replaced with new ones of similar economic status, geographic characteristics and population size from the same stratum due to administrative changes and practical considerations. Since 2009, the unit of surveillance in urban areas has changed to districts while that in rural areas remained at the county level.

Webappendix 3. Additional details on sample size calculation of MCHSS

In 1991, the desired sample size was calculated using the following formula:

$$N = \frac{(\frac{Z}{d})^2 * r * (1-r)}{b}$$

where *Z* is the *Z* value, which is approximately 1.96. *d* is the desired margin of error, which was set at 2.5%. *r* is the estimated infant mortality. *b* is the estimated crude birth rate. Both infant mortality and birth rate estimates were adopted from the 1982 national census.

Since 1996, design effect has been taken into consideration when calculating the desired sample size for each stratum, that is

$$N = \frac{(\frac{Z}{d})^2 * r * (1-r)}{b} * deff$$

where *deff* is the design effect, which was assumed to be 2.0. Infant mortality and crude birth rates were based on National Bureau of Statistics estimates. A similar formula was used for sample size calculation when MCHSS expanded in 2006, with different sources of input data as explained in the main text.

Webappendix 4. MCHSS sampling

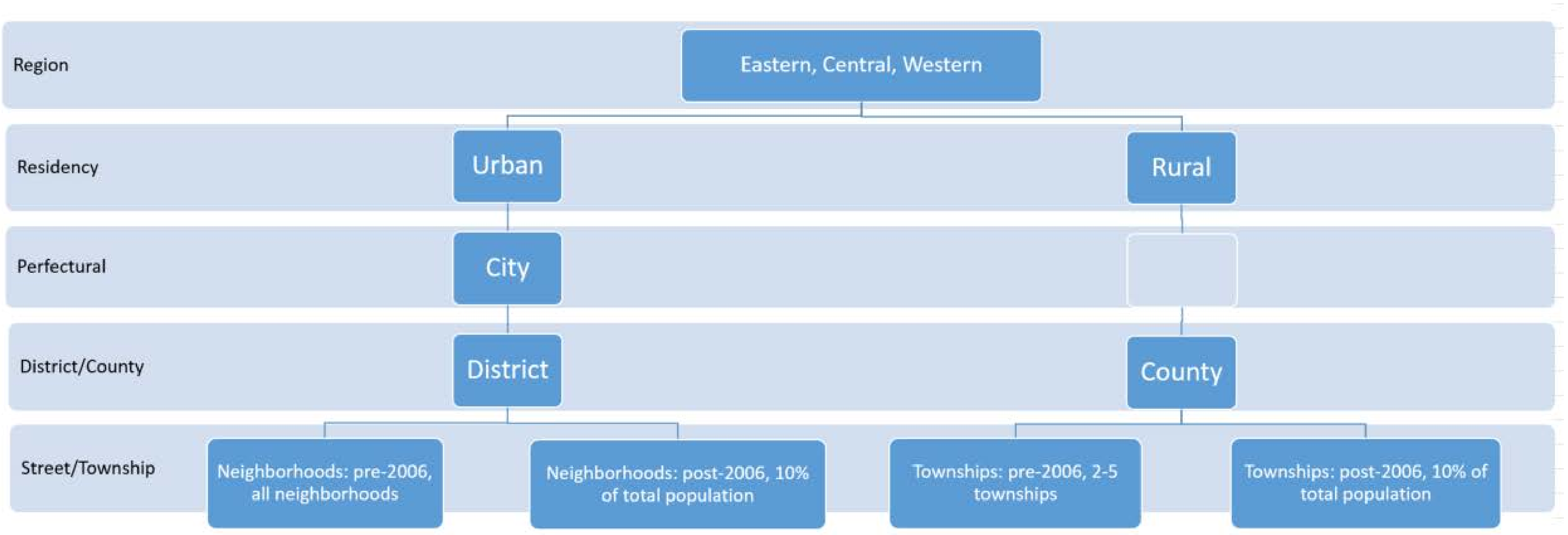

Webappendix 5. Child Death Registration Card

Child Death Registration Card

__________District/County☐☐☐☐☐☐

No. ☐☐☐☐☐☐☐☐☐

Address:____Township/District____Street/Village

Father's name:_______ Mother's name_______

Child's name:________

(1) Registered permanent residence (2) Non-local registered permanent residence for less than one year (3) Non-local registered permanent residence for more than one year ☐

Sex: 1.Male 2.Female ☐

Date of birth:

Year		Month		Day	

Birth weight: ______g

(1) measured (2) estimated ☐

Gestational weeks: ______weeks

Place of birth:

(1) provincial/municipal hospital (2) district/county hospital (3) community/township health center (4) village clinic (5) on the way (6) in home ☐

Date of death:

Year		Month		Day	

Age at death: ___years___months___days

Cause of death:

(a) Disease or condition directly leading to death

____

(b) Disease or condition leading to (a)____________

(c) Disease or condition leading to (b)____________

(d) Disease or condition leading to (c)____________

Underlying cause of death____________________

Classification Code ☐☐

ICD-10 code ☐☐☐☐☐

Place of death: (1) hospital/clinic (2) on the way (3) in home ☐

Premortality treatment: (1) inpatient treatment (2) outpatient treatment (3) without treatment ☐

Level of hospital: (1) provincial/municipal hospital (2) district/county hospital (3) community/township health center (4) village clinic (5) without treatment ☐

Reason for untreated: (Single Choice)

(1) financial hardship (2) traffic inconvenience (3) time limited (4) parents unaware of the serious condition (5) manners and customs (6) others (specify) ☐

Basis for diagnosis: (1) pathological autopsy (2) clinical diagnosis (3) inference ☐

Basis or evidence to infer the causes of death: (Please describe in details, using the blank on the back.)

____

____

Report unit____________ Name of reporter__________ Date of report___________

Classification Codes for causes of death

01 dysentery
02 sepsis
03 measles
04 tuberculosis
05 other infectious and parasitic diseases
06 leukemia
07 other neoplasms
08 meningitis
09 other diseases of the nervous system
10 pneumonia
11 other diseases of the respiratory system
12 diarrhea
13 other diseases of the digestive system
14 congenital heart diseases
15 neural tube defects
16 Down's syndrome
17 other congenital malformations
18 premature delivery or low birth weight
19 birth asphyxia
20 tetanus of newborn
21 scleredema of newborn
22 intracranial hemorrhage
23 other diseases of newborn
24 drowning
25 traffic accident
26 accidental suffocation
27 accidental poisoning
28 accidental falls
29 other accidental injuries
30 endocrine, nutritional and metabolic diseases
31 diseases of blood and blood-forming organs
32 diseases of the circulatory system
33 diseases of the urinary system
34 other diseases
35 ill-defined and unknown causes of death

Webappendix 6. The distribution of deaths by cause ascertaining method by age and year (unweighted), MCHSS, China

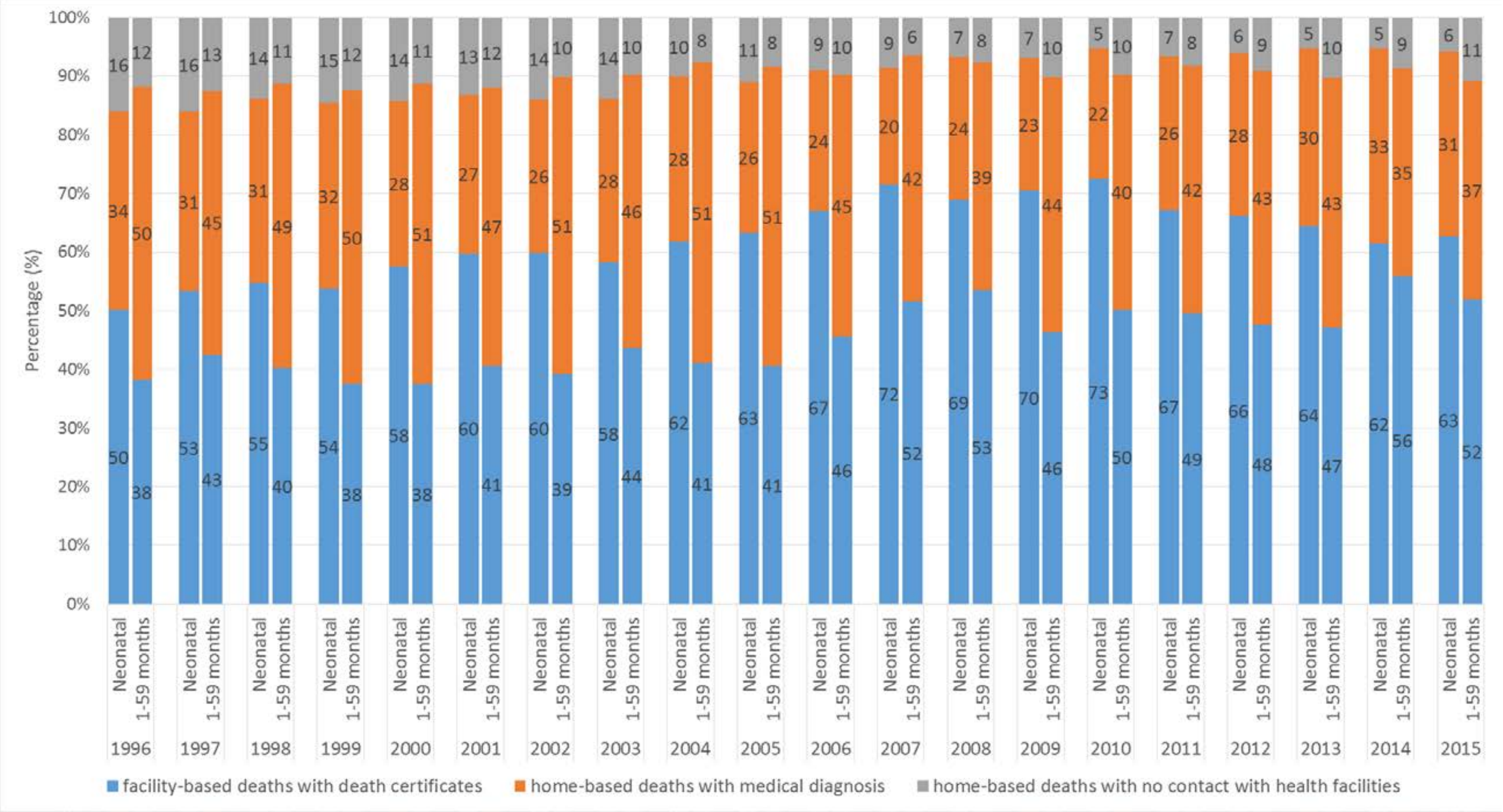

Webappendix 7. Additional details on quality control of MCHSS

During data triangulation and cross validation across multiple local sources, a number of techniques are used to improve the completeness of births and deaths reporting. These include focus group discussion with village doctors, household visits, medical records review of livebirths and stillbirths, pregnancy and delivery history, Apgar scores for newborns, review of civil registrations records, vaccination records and history, and records from Family Planning Offices, Centers of Disease Control, Public Security Bureaus, Civil Affairs Bureaus, and New Rural Cooperative Medical Scheme Service Offices, etc.[3]

The under-reporting rates are calculated based on the annual quality control study results using the following formula:

$$Underreporting\ rate = N_{missed} / (N_{reported} + N_{missed}) \times 100\%$$

where N_{missed} is the number of missed livebirths or deaths identified during the annual quality control study, and $N_{reported}$ is the number of livebirths or deaths originally reported to MCHSS.

Since 2010, neonatal death audit has been implemented in MCHSS in health departments and maternal and child health facilities at and above the district/county level to validate causes of neonatal deaths and improve neonatal survival. Local health administrative authorities establish a panel of experts that include at least two neonatologists/pediatricians, two obstetricians, health and hospital administrative staff, and MCHSS staff. The expert panel meets with physicians providing care for the deceased neonates, review and adjudicate causes of deaths, and issue review reports for all neonatal deaths occurred in health facilities in MCHSS. All deaths are audited at district/county level every six months, and difficult cases are further audited at the city and provincial level once a year. Experts also collect information on main factors contributing to neonatal deaths, determine whether the deaths are either preventable, preventable under certain conditions, or inevitable, and provide suggestions on interventions to prevent similar neonatal deaths in the future. Annual reports of neonatal death audit are submitted to MCHSS National Office and National Center for Women and Children's Health in China Centers of Disease Control.[3]

Webappendix 8. Causes of deaths categorization and mapping between MCHSS classification, ICD-10, and the Child Health Epidemiology Reference Group (CHERG) classification

Cause name (CHERG)	ICD-10 code (CHERG)	Cause name (MCHSS)	ICD-10 (MCHSS)
All causes	A00-Y89	All causes	A00-Y89
**I. Communicable, maternal, neonatal and nutritional conditions***	A00-B99, D50-D53, D64.9, E00- E02, E40-E64, G00, G03-G04, H65-H66, J00-J22, J85, N30, N34, N39.0, N70-N73, O00-P96, U04		
HIV/AIDS	B20-B24	05 Other infectious and parasitic diseases	B20-24
Diarrhea diseases	A00-A09	01 Dysentery	A09.0, A03.9
		12 Diarrhea	A00-A09
Pertussis	A37	05 Other infectious and parasitic diseases	A37
Tetanus	A33-A35	20 Tetanus of newborn	A33
		05 Other infectious and parasitic diseases	A34-A35
Measles	B05	03 Measles	B05
Meningitis/Encephalitis	A39, A83, A84-A87, G00, G03, G04	05 Other infectious and parasitic diseases	A39.1-A39.9, A83, A84-A87, G03, G04
		08 Meningitis	A39.0, G00
Malaria	B50-B54, P37.3 , P37.4	05 Other infectious and parasitic diseases	B50-B54
		23 Other diseases of newborn	P37.3, P37.4
Pneumonia	H65-H66, J00-J22, J85, P23	10 Pneumonia	J09-10.0, J11.0, J12-18, P23
		11 Other diseases of respiratory system	J00-J08, J10-11 (excluding J10.0, J11.0), J19-J22, J85
		34 Other	H65-H66
Preterm birth complications	P01.0, P01.1, P07, P22, P25-P28, P61.2, P77	23 Other diseases of newborn	P01.0, P01.1, P22, P25-28, P61.2, P77
		18 Premature delivery or low birth weight	P07
Intrapartum related complications	P01.7-P02.1, P02.4-P02.6, P03, P10-P15, P20-P21, P24, P50, P90-P91	19 Birth asphyxia	P01.7-P02.1, P02.4-P02.6, P03, P20-P21, P24
		22 Intracranial hemorrhage	P10
		23 Other diseases of newborn	P11-15, P50, P90-P91

		10 Pneumonia	P24
Sepsis and other infectious conditions of the newborn	P35-P39 (excluding P37.3, P37.4)	23 Other diseases of newborn	P35-P39 (excluding P37.3, P37.4)
		02 Sepsis	P36
Other Group I	Remainder(A10-A36, A38, A40-A82, A88-A99, B00-B04, B06-B19, B25-B49, B55-B99, D50-D53, D64.9, E00-E02, E40-E64, J00-J22, N30, N34, N39.0, N70-N73, O00-O99, P01.2-P01.6, P02.2-P02.3, P02.7-P02.9, P04-P06, P08-P09, P16-P19, P29-P34, P40-P49, P51-P61.1, P61.3-P76, P78-P96, U04)	02 Sepsis	A40-A41
		04 Tuberculosis	A15-19, B90
		05 Other infectious and parasitic diseases	A20-A32, A36-A38, A42-A82, A88-A99, B00-B04, B06-B19, B25-B49, B55-B99 (excluding B90)
		31 Diseases of blood and blood-forming organs	D50-D53,D64.9
		30 Endocrine, nutritional and metabolic diseases	E00-E02, E40-E64
		10 Pneumonia	J84.9
		33 Diseases of the urinary system	N30, N34, N39.0
		34 Other diseases	N70-N73, O00-O99, U04
		18 Premature delivery or low birth weight	P05
		21 Scleredema of newborn	P80.0,P83.0
		22 Intracranial hemorrhage	P52
		12 Diarrhea	P78.3
		23 Other diseases of newborn	P01.2-P01.6, P02.2-P02.3, P02.7-P02.9, P04, P06, P08-P09, P16-P19, P29-P34, P40-P49, P51, P53-P61.1, P61.3-P76, P78-P96 (excluding P78.3, P80.0 and P83.0)
**II. Non-communicable diseases**	C00-C97, D00-D48, D55-D64 (excluding D 64.9) , D65-D89, E03- E34, E65-E88, F01-F99, G06- G98, H00-H61, H68-H93, I00- I99, J30-J84, J86-J98, K00-K92, L00-L98, M00-M99, N00-N28, N31-N32, N35-N64 (excluding N39.0) , N75-N98, Q00-Q99		

Congenital abnormalities	Q00-Q99	14 Congenital heart disease	Q20-24
		15 Neural tube defects	Q00-01,Q05
		16 Down's syndrome	Q90
		17 Other congenital malformations	Q02-04, Q06-18, Q25-89,Q91-99
Other Group II	Remainder (C00-C97, D00-D48, D55-D64 (excluding D 64.9) , D65-D89, E03- E34, E65-E88, F01-F99, G06- G98, H00-H61, H68-H93, I00- I99, J30-J84, J86-J98, K00-K92, L00-L98, M00-M99, N00-N28, N31-N32, N35-N64 (excluding N39.0) , N75-N98,)	06 Leukemia	C91-95
		07 Other neoplasms	C00-D48 (excluding C91-95)
		31 Diseases of blood and blood-forming organs	D55-D89 (excluding D64.9)
		30 Endocrine, nutritional and metabolic diseases	E00-E34, E65-E88
		34 Other diseases	F01-F99,H00-H61, H68-H93 , L00-L98, M00-M99
		09 Other diseases of the nervous system	G06-G98
		11 Other respiratory diseases	J30-J84, J86-J98
		12 Diarrhea	K50-52, K58.0, K59.1
		13 Other diseases of the digestive system	K00-93 (excluding K50-52, K58.0, K59.1)
		33 Diseases of the urinary system	N00-N28, N31-N32, N35-N64 (excludingN38.0), N75-N98
		32 Diseases of the circulatory system	I00- I99
**III. Injuries**	V01-Y89	24 Drowning	W65-W74
		25 Traffic accident	V01-V99
		26 Accidental suffocation	W75-W84
		27 Accidental poisoning	X40-X49
		28 Accidental falls	W00-W19
		29 Other accidental injuries	V01-Y98, (excluding W00-19, W65-84, V01-99, X40-X49)

Webappendix 9. Sampling probability (%) by region-and-residency strata in MCHSS

Region	Residency	1996	2000	2010
East	Urban	4.6	3.8	5.4
East	Rural	0.6	0.4	1.9
Central	Urban	2.7	2.4	3.1
Central	Rural	0.4	0.4	2.1
West	Urban	5.3	4.8	6.6
West	Rural	0.7	0.9	4.2

Webappendix 10. GATHER checklist

Item	Checklist item*	Section(s) or sources providing information
Objectives and funding		
1	Estimated indicator and population	Methods
2	Funding sources	Funding section of summary
Data Inputs		
For all data inputs from multiple sources that are synthesized as part of the study:		
3	Data identification	Methods and appendix 1-4
4	Inclusion/exclusion criteria	Not applicable
5	Included data sources and their main characteristics	Methods and appendix 1-4
6	Potential important biases of input data	Discussion and appendix 2-3, 7-9
For data inputs that contribute to the analysis but were not synthesized as part of the study:		
7	Source of other data inputs	Methods
For all data inputs:		
8	Accessible input data files	Open access databases**
Data analysis		
9	Conceptual overview of the data analysis method	Methods and open access databases**
10	Description of all steps of the analysis	Methods and appendix 7-9
11	Model selection methods	Not applicable
12	Model performance and/or sensitivity analysis.	Not applicable
13	Uncertainty estimation methods	Methods and discussion
14	Statistical code	Open access databases**
Results and discussion		
15	Accessible estimates data files	Open access databases**
16	Uncertainty of the estimates	Results and discussion
17	Results interpretation in light of existing evidence	Results and discussion
18	Limitations of the estimates	Discussion

*Detailed GATHER statement with explanation and elaboration of the items could be found on gather - statement.org

** Open access databases with input files and analytical code of the study could be accessed on the Maternal and Child Epidemiology Estimates project website: http://tinyurl.com/Hopkins-MNCH-Chinacod-openacce

Webappendix 11. Cause specific mortality fractions by age among children under-five years of age in China in 2015

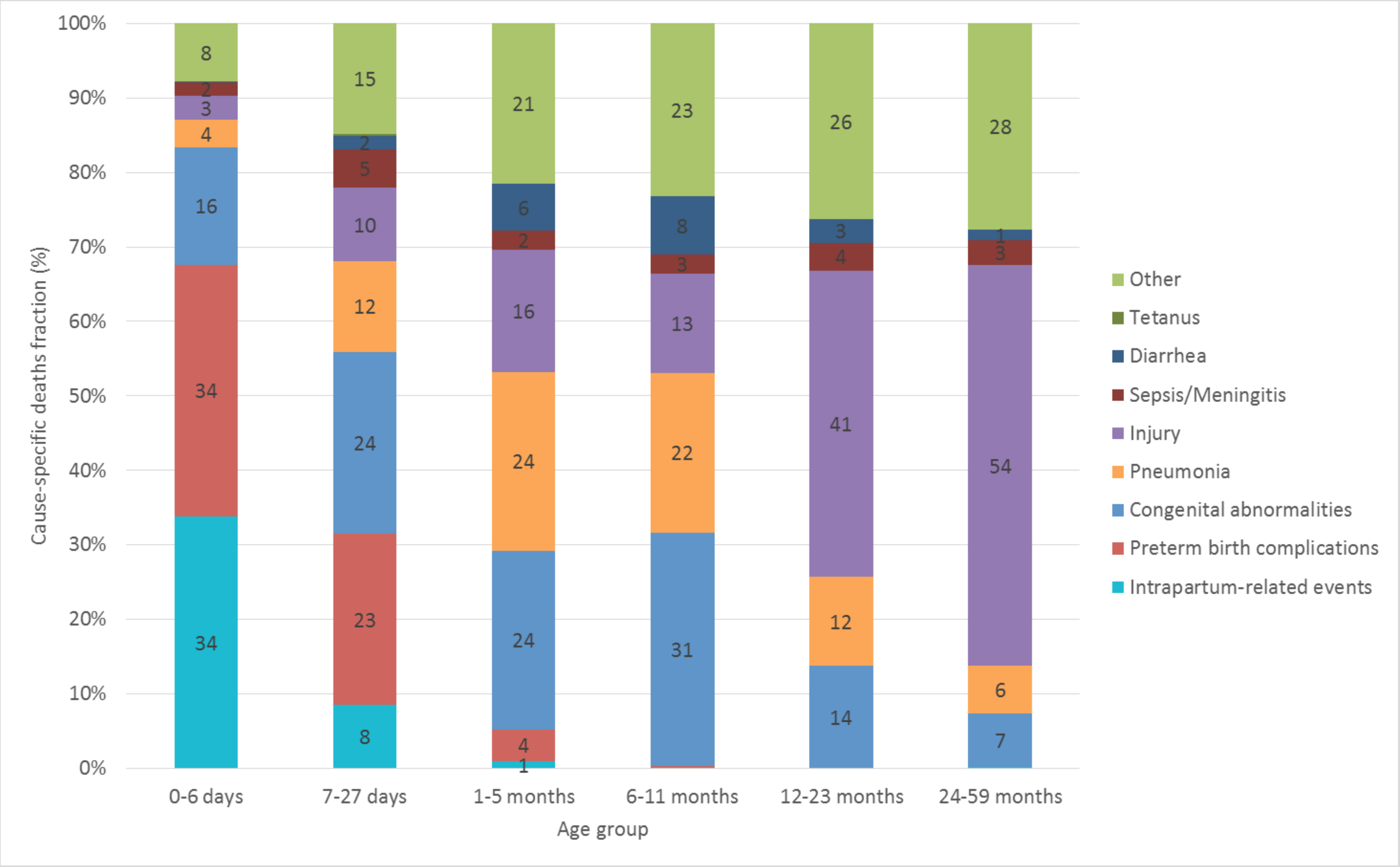

Webappendix 12. Cause specific mortality fractions by residency and region in China in 2015

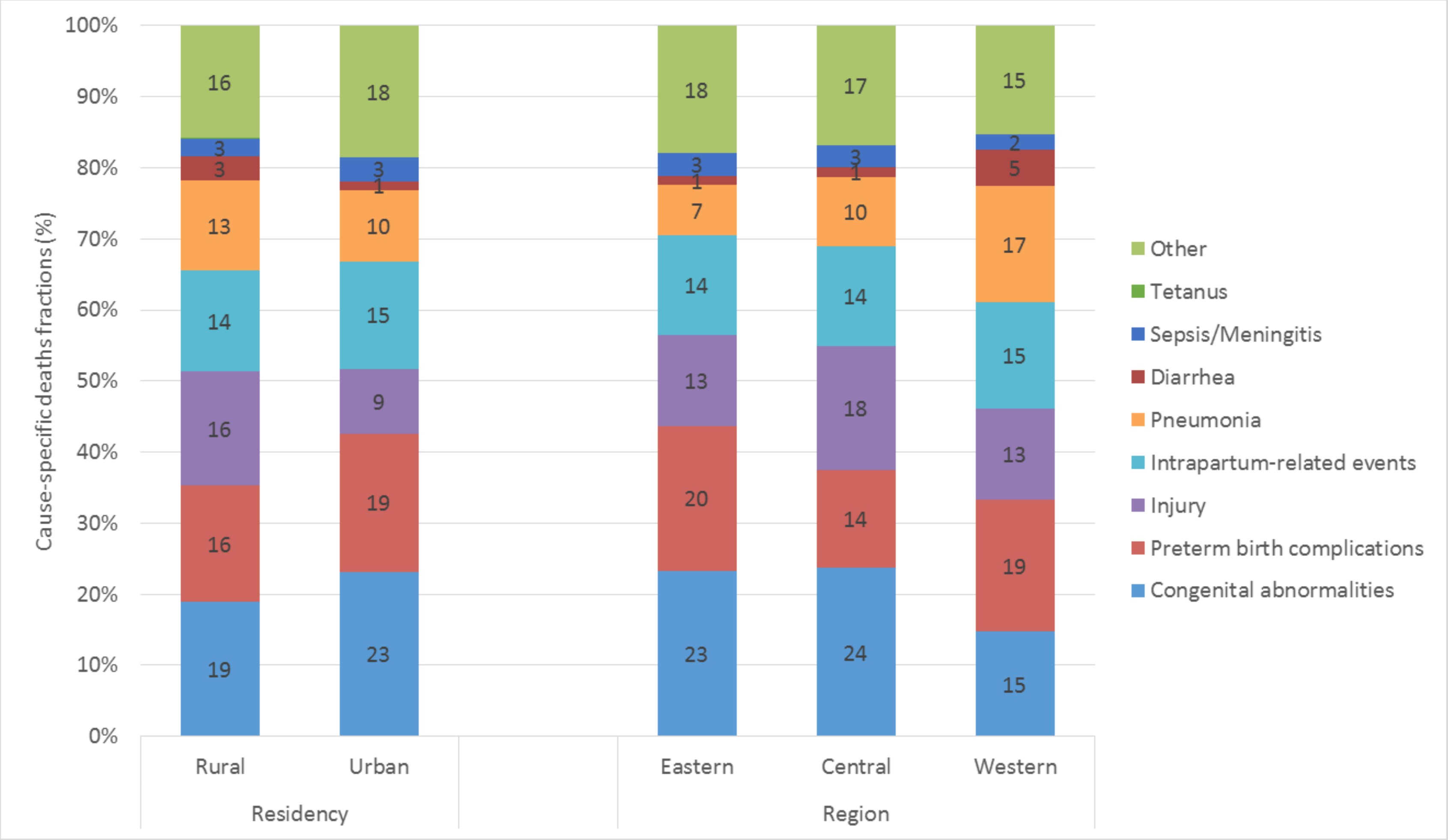

Webappendix 13. Comparison of age-and-cause-specific mortality fractions in 2015 between He et al. and Song et al.

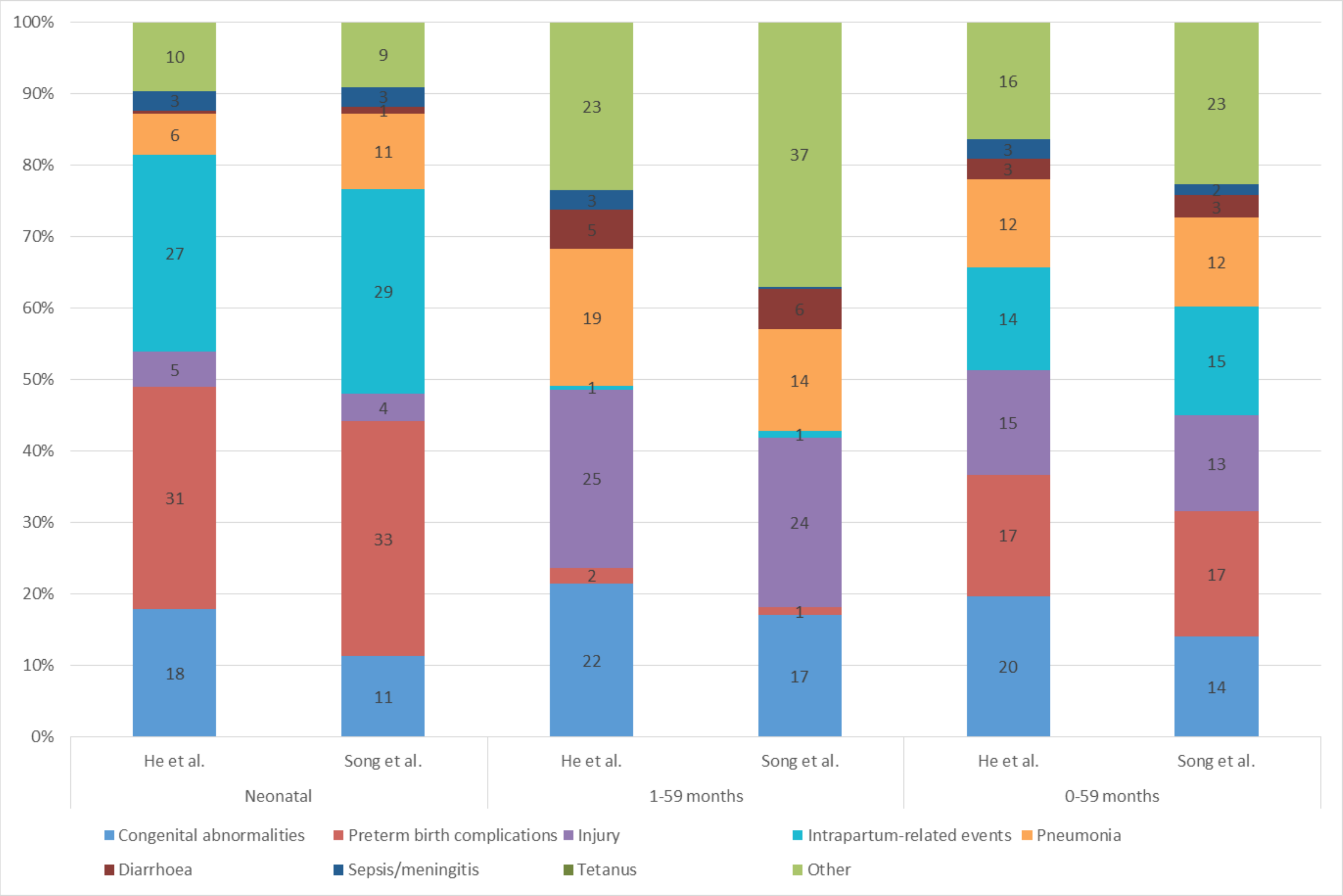

Webappendix 14. Comparison of trends in national U5MR and NMR by estimation sources, China, 1996-2015

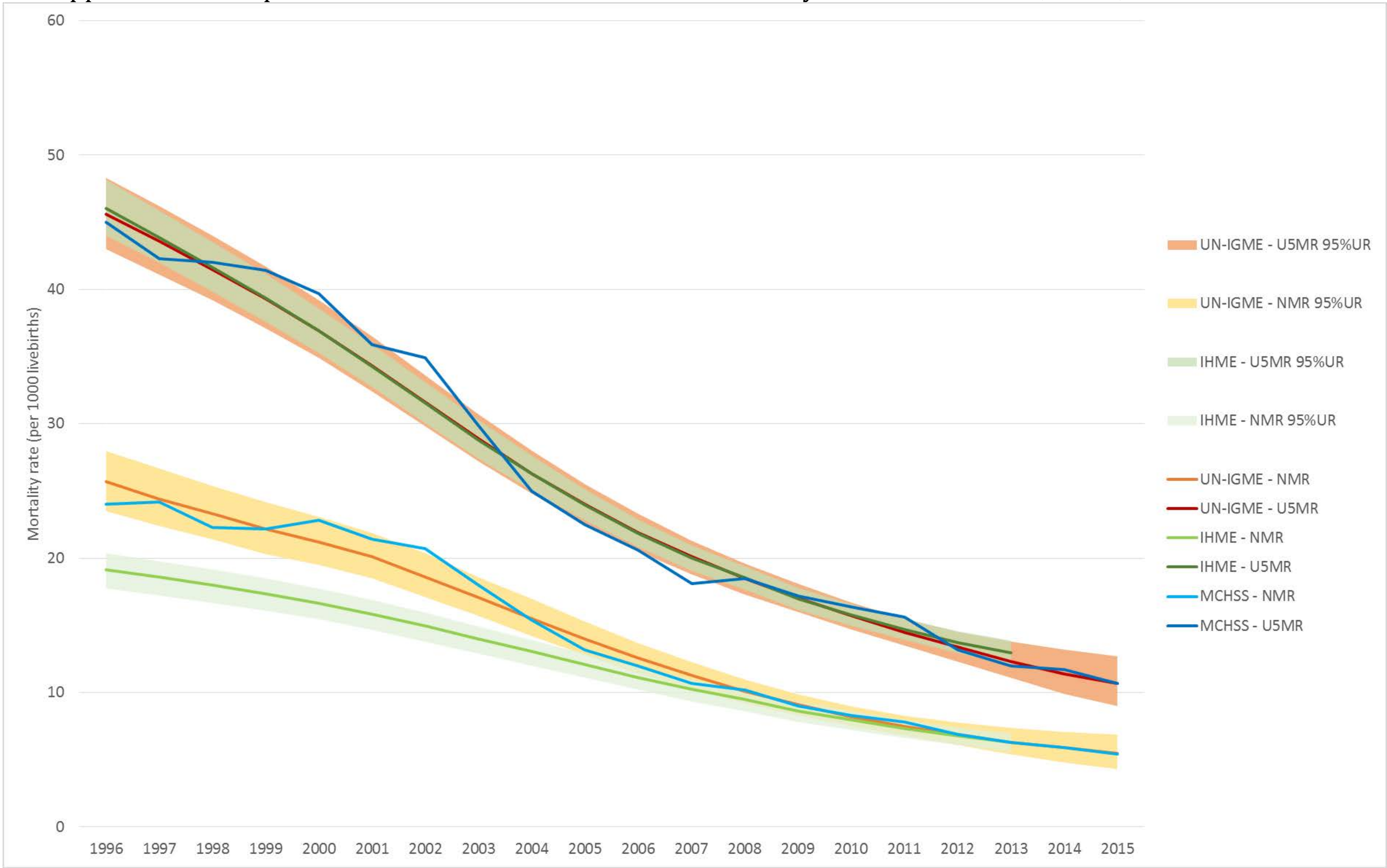

UN-IGME: United Nations' Interagency Group on Child Mortality Estimation[4]; IHME: Institute of Health Metrics and Evaluation[5]

References

1. Feng S, Shi X. Sampling Survey - Theory, Method and Practice. Shanghai, China: Shanghai Scientific and Technical Publishers; 1996.
2. Wang YP, Miao L, Dai L, et al. Mortality rate for children under 5 years of age in China from 1996 to 2006. *Public Health* 2011; **125**(5): 301-7.
3. National office of China Maternal and Child Health Surveillance System. Handbook of Maternal and Child Health Surveillance System in China, 2013 version: National office of China Maternal and Child Health Surveillance System, 2013.
4. You D, Hug L, Ejdemyr S, et al. Global, regional, and national levels and trends in under-5 mortality between 1990 and 2015, with scenario-based projections to 2030: a systematic analysis by the UN Inter-agency Group for Child Mortality Estimation. *The Lancet* 2015 ; **386**(10010): 2275-86.
5. Wang H, Liddell CA, Coates MM, et al. Global, regional, and national levels of neonatal, infant, and under-5 mortality during 1990–2013: a systematic analysis for the Global Burden of Disease Study 2013. *The Lancet* 2014; **384**(9947): 957-79.
